# Genetic insights into hepatitis E virus through environmental surveillance in Europe

**DOI:** 10.1016/j.onehlt.2025.101302

**Published:** 2025-12-15

**Authors:** Hao Wang, Marianela Patzi-Churqui, Linn Dahlsten Andius, Kristina Nyström, Martin Lagging

**Affiliations:** a Department of Infectious Diseases, Institute of Biomedicine, University of Gothenburg, Gothenburg, Sweden; b Department of Clinical Microbiology, Sahlgrenska University Hospital, Gothenburg, Sweden

**Keywords:** HEV, Wastewater, Genotyping, One health, Animal reservoir, Zoonotic transmission

## Abstract

Zoonotic hepatitis E has been a growing public health concern in Europe, but the transmission of its causative agent, hepatitis E virus (HEV), remains incompletely understood. Environmental surveillance, particularly through wastewater monitoring, has proven valuable for tracking viral circulation and variant shift during the COVID-19 pandemic, yet its application to HEV is still limited. In this review, we systematically analyzed HEV sequences across Europe, focusing on environmental sources from a genetic perspective. Of more than 13,100 HEV sequences deposited in the NCBI database, only 2.4 % (316/13,118) originated from environmental samples, including wastewater, surface water, and biosolids. Additional typing data from the literature revealed highly uneven geographic distribution, with 97 % of environmental sequences reported from Italy, France, the United Kingdom (UK), Spain, Sweden, and Germany. HEV-3 was the dominant genotype, while HEV-1 and HEV-4 were occasionally detected. Subtypes 3c and 3f were most common, but their prevalence varied across countries and sample types. Some countries, such as France, Sweden, and the UK, exhibited divergent subtype patterns between humans, animals, and environmental sources, whereas others, such as Spain and Germany, showed more consistent distributions. These findings highlight the importance of integrating clinical, veterinary, and environmental surveillance to better understand HEV transmission in Europe under a One Health framework. However, the scarcity of environmental data, technical challenges in sequencing, and lack of standardized protocols limit comprehensive assessment of HEV circulation. Expanding sequencing efforts, improving detection methods, and coordinating international surveillance frameworks will be critical to strengthen HEV monitoring and preparedness against emerging HEV threats.

## Highlights

• HEV environmental sequencing data in Europe remains critically limited.
• Uneven geographic distribution in HEV surveillance across Europe.
• Substantial variation in HEV-3 subtypes across European countries and sample types.
• Technical challenges hinder accurate HEV genotyping from environmental sources.
• Archived wastewater samples from COVID-19 surveillance offer a valuable resource.

## Graphical abstract

**Figure:**
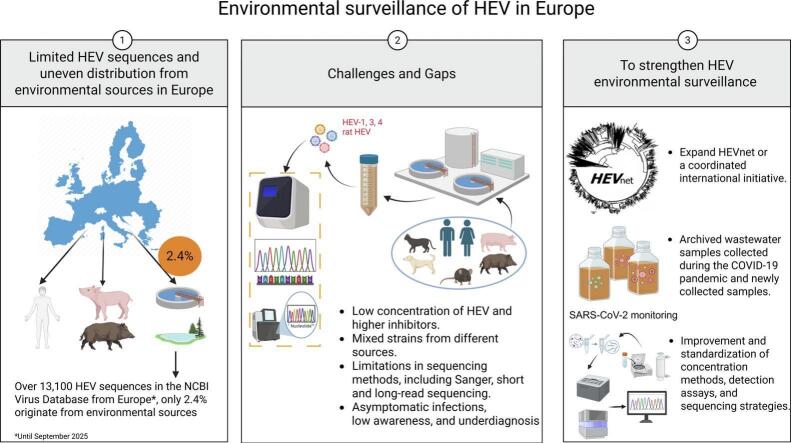

## 1 Introduction

Hepatitis E virus (HEV) is a major cause of acute viral hepatitis globally, with increasing reports in Europe in recent years [1,2]. The predominant genotype in Europe is HEV-3, circulating widely among humans and various animal reservoirs, mainly domestic pigs and wild boars. Transmission of HEV-3 occurs primarily through zoonotic routes, such as the consumption of undercooked pork and game meat. Alternative transmission routes in industrialized countries include blood transfusion [3], while waterborne transmission is also suspected [4,5]. Sporadic HEV-4 infections have been reported in several European countries, such as Belgium, Italy, Hungary, and France [[6], [7], [8]], while HEV-1 infections are mostly travel-associated [2]. Despite increasing clinical awareness and improved diagnostics, our understanding of HEV transmission in Europe remains incomplete.

Environmental surveillance, which involves monitoring viruses in sources like wastewater, surface water, and soils, has served as a complementary tool for monitoring viral circulation for decades. During the COVID-19 pandemic, this approach, particularly wastewater-based epidemiology, gained prominence by providing timely insights into the spread and variant shifts of SARS-CoV-2 at the community level, demonstrating its value for public health [[9], [10], [11]]. Post-pandemic, this approach has expanded to non-enteric viruses like influenza viruses, respiratory syncytial virus, and mpox [[12], [13], [14]]. However, its application for HEV is relatively underexplored and less routine compared to other human enteric viruses, such as norovirus and adenovirus.

A prior review has assessed the prevalence of HEV in different water matrices worldwide, and noted HEV is widely presented in environmental waters [15]. Environmental sources likely play a critical role in understanding HEV dissemination. Notably, rat HEV, a distinct species capable of causing acute and chronic hepatitis in humans, has also been detected in wastewater [[22], [23], [24], [25], [26]], adding complexity to environmental HEV epidemiology. Yet, methodological challenges such as low viral concentrations, genetic diversity, and the complexity of environmental matrices limit the comparability and interpretation of these studies. More studies are needed to assess the full potential of environmental sources in the understanding of HEV spread.

Although HEV is a global concern, Europe represents a special context for studying environmental surveillance. Unlike HEV-1 and HEV-2, which are waterborne transmitted in low- and middle-income countries, the role of environmental waters in the spread of HEV-3, the dominant genotype in Europe, is not well understood. Previous reviews have summarized the HEV occurrence in water matrices or discussed general challenges in HEV detection and environmental surveillance [15,27,28]. However, none have examined regional differences in genotype or subtype distribution across humans, animals, and environmental sources. This review addresses this gap by systematically analyzing HEV sequences from environmental samples and comparing them with human and animal data in Europe. This One Health approach will deepen our understanding of HEV transmission across Europe and guide practical use of integration environmental data into epidemiology efforts.

## 2 Materials and methods

### 2.1 Data collection

All available HEV sequence data were retrieved from the National Center for Biotechnology Information (NCBI) Virus database (https://www.ncbi.nlm.nih.gov/labs/virus/vssi/). The search was performed on September 10, 2025, using the terms “Virus/Taxonomy: Hepatitis E Virus (taxid: 291484) and Orthohepevirus (taxid: 1678141) and Paslahepevirus balayani (taxid: 1678143)” and “Geographic Region: Europe.” This search yielded 13,118 sequences. For each sequence, metadata including accession number, sequence length, country, tissue/specimen/source, host, and collection date were extracted for further analysis. Sequences were grouped by country and categorized as human, animal, environment, or others. A total of 836 sequences lacked source information in the database. Two researchers independently verified these records by checking the original publications or corresponding GenBank entries. Sequences without clear source information or derived from cell culture experiments were classified as “Others”. It is noteworthy that some laboratories in Europe deposit their sequences in the HEVnet database rather than the NCBI database. However, because HEVnet data are access-restricted and require permission for use, these sequences were not included in the present analysis.

### 2.2 Literature review

To obtain additional HEV sequences from environmental sources but not deposited in NCBI, a systematic literature search was conducted between September 15 and September 19, 2025, in PubMed and Google Scholar. The search strategy combined “Hepatitis E virus,” “HEV,” “Viral hepatitis E,” or “Paslahepevirus balayani” with environmental keywords (“water,” “wastewater,” “sewage,” “soil,” “environmental source”) and “genotype,” together with the name of each European country. The inclusion criteria included: a) only studies conducted in Europe and reported in English; b) reporting HEV sequences, genotyping data, or subtype assignments. Those studies that do not report any genotyping or sequence information, and unpublished datasets or grey literature were excluded. Duplicates between NCBI sequences and published studies were identified by accession number and removed. After manual screening, 40 European studies were identified. From these, 490 sequences or genotyping results were extracted. Of these, 316 sequences were already deposited in NCBI, while 174 were reported only in the original publications. Extracted information included country, source type, sampling year, and genotyping results. All data are summarized in Table 1.

**Table 1:** Summary of studies and HEV sequences obtained from environmental samples across Europe.

Countries	Source	Sampling collection period	Genotyping											Total^2^	Ref
			HEV1	HEV4	3a	3b	3c	3i	3e	3f	3h	3m	Unclassified		
Sweden	wastewater	2023				1	13							14	[24]
Sweden	wastewater	2016–2018					6							6	[29]
Sweden	wastewater	2017					4			1				5	[34]
Sweden	water from drinking water treatment plant	2016–2017			1		7							8	[4]
France	wastewater	2014–2015								6				6	[35]
France	wastewater	2013								1				1	[36]
France	sewage	1998											1	1	[37]
France^1^	wastewater and rivers water	2023					2					2		4	[23]
France	wastewater	2013–2014								2				2	[38]
France^1^	wastewater	2006–2016								3				3	[39]
France	wastewater and effluents from slaughterhouse	2016–2017					2	1						3	[40]
France^1^	wastewater	2023–2024					54			31	1			86	[41]
Serbia	river water	2013											2	2	[19]
Slovenia	surface water	2004–2005											2	2	[42]
Norway	wastewater	2008–2009	1										3	4	[43]
Portugal	wastewater	2013						1		1				2	[44]
Russia	sewage from pig farm	2012, 2014											2	2	[45]
Switzerland	wastewater	2010–2011	1											1	[46]
Germany	wastewater, surface water	2014–2019					10			2			3	15	[16]
Germany^1^	wastewater, and environmental samples	2020–2021					8							8	[47]
Netherlands	river water	2004–2005							1	1				2	[48]
UK	sewage	2014–2015			1		52		2	2			4	61	[20]
UK^1^	wastewater	2019–2020					5		4			1		10	[49]
Italy^1^	sewage	2011–2019	18							3			56	77	[50]
Italy	sewage	2016–2017					4			1				5	[51]
Italy	seawater and water samples	2015											5	5	[52]
Italy	sewage and surface water	2011								3			3	6	[53]
Italy	sewage	2008–2009	18										1	19	[54]
Italy	sewage and river water	2013											2	2	[55]
Italy^1^	wastewater	2022		1 (4d)										1	[21]
Italy	Pig slurry	2015								8			15	23	[17]
Italy	sewage	2012–2016	13										25	38	[56]
Italy	environmental water	2012–2017											1	1	[18]
Spain	sewage	2021–2023								1				1	[26]
Spain	sewage	2017								3				3	[57]
Spain	sewage	2000–2008	1										14	15	[58]
Spain	sewage, biosolids, and sludge	2001, 2003, 2004–2007	6										19	25	[59]
Spain	sewage and biosolids	2004–2006	1										4	5	[60]
Spain	sewage	1994–2002	1										14	15	[37]
Spain	sewage	1994–1998	1											1	[61]

1 Studies utilizing short- and long-read sequencing techniques.

2 The analysis was based on data available until September 2025.

### 2.3 Genotyping and phylogenetic analysis

Six European countries, including France, the UK, Italy, Spain, Germany, and Sweden, with at least 20 environmental HEV sequences were included for comparison of genotype and subtype distribution among humans, animals, and environmental sources. To ensure accurate genotyping, only sequences longer than 300 bp were retrieved from the NCBI database. The genotyping analysis for Sweden had been previously reported [29] and was included in the comparison. In total, 365 sequences from the UK, 693 from Germany, 750 from Spain, 758 from Italy, and 2912 from France were downloaded. The sequences were categorized as originating from either humans (*Homo sapiens*) or animals. Genotyping was performed using the Hepatitis E Virus Genotyping Tool (https://mpf.rivm.nl/mpf/typingtool/hev/), and the results from the “phylo minor” output (Supplementary Table 1), which provides subtype-level classification, were used for subsequent analyses.

The RNA-dependent RNA polymerase (RdRp) region of open reading frame 1 (ORF1) and the capsid region of ORF2 are the two genomic regions most frequently used for HEV genotyping in EU/EEA reference laboratories [30]. Reported studies investigating HEV in environmental sources used both regions in roughly equal proportions. For consistency and comparability across datasets, we therefore selected HEV sequences of the RdRp region from humans, animals, and environmental sources for phylogenetic analysis. Reference strains representing HEV genotypes 1–8 were included for accurate classification. In total, 957 sequences were analyzed. Multiple sequence alignment based on 309 bp of the RdRp region was performed using MAFFT version 7 online platform and was provided in supplementary materials [31]. Phylogenetic trees were constructed in MEGA11 using the unweighted pair-group method with arithmetic mean (UPGMA) and 1000 bootstrap replicates [32]. Tree annotation and visualization were carried out in iTOL version 7.2.2 [33].

## 3 Results

**Fig. 1:**
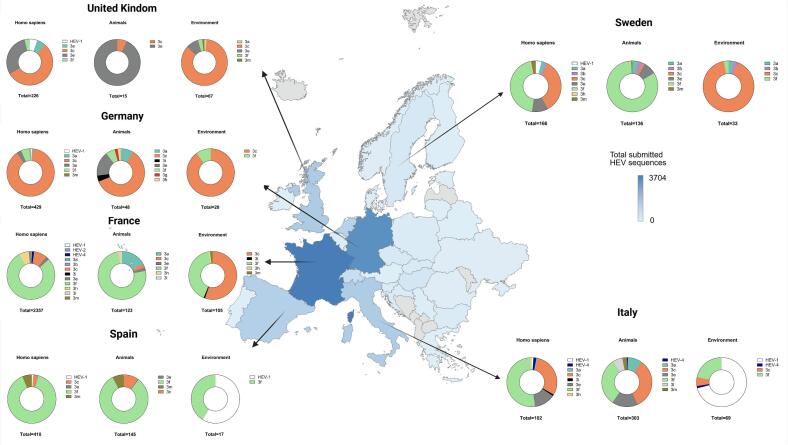
Geographical distribution of HEV sequences in Europe. The map of Europe illustrates the total number of HEV sequences submitted to the NCBI database by each country up to September 2025, with color intensity reflecting the number of sequences. Pie charts for France, UK, Italy, Spain, Germany, and Sweden detail the genotype and subtype distribution of HEV sequences from *Homo sapiens* (human), animals, and environmental samples, excluding unclassified sequences.

The analysis of HEV sequences from the NCBI Virus Database revealed significant variation across European countries and source types (Fig. 1 and Supplementary Table 2). As of September 2025, a total of 13,118 sequences from 31 European countries have been deposited. Most sequences (9202; 70.1 %) are from humans, followed by various animals (3552; 27.1 %), including pigs, wild boars, rodents, deer, and moose. In contrast, only 316 HEV sequences (2.4 %) are from environmental sources, such as wastewater, surface water, and biosolids, and 46 sequences from other origins or unknown sources. France and Germany had most sequences with 3704 and 3106, respectively, predominantly from humans (3298 and 2502; Supplementary Table 2). Italy showed a more even distribution across sources, with 214 human, 730 animal, and 112 environmental sequences. Almost half of the European countries (15/31) reported less than 50 HEV sequences, which indicates regional differences in sampling efforts.

Only ten European countries have reported HEV sequences from environmental sources (Supplementary Table 2). Among them, six countries reported more than 10 sequences, with Italy (112), the United Kingdom (UK; 71), and Spain (65) contributing the most, while much of Europe remains unrepresented. Although with a limited number of sequences, there have been annual increases in detection post-2013, mainly from Italy, UK, Sweden, Germany, and France, which suggests growing but uneven application of environmental monitoring across Europe. However, reports have been sporadic since the COVID-19 pandemic.

To capture additional data, sequences reported in the literature but not deposited in the NCBI Virus database were also analyzed. In total, 40 European studies were identified, with Italy contributing the most (10 studies; Table 1), followed by France (8 studies) and Spain (7 studies). Most investigations focused on wastewater (33 studies), while only three targeted solid samples. Sampling covered the period 1994–2024. Reported HEV prevalence was generally higher in influent wastewater (sewage) than in other environmental matrices but varied substantially across countries and time periods, ranging from 1 % in Spain (2021−2023) to more than 90 % in the United Kingdom (2014–2015), France (2023–2024), and Sweden (2016–2018). Sequencing approaches were predominantly based on Sanger sequencing, mainly targeting either ORF1 or ORF2, while seven studies applied advanced sequencing methods, including both short- and long-read sequencing (Table 1).

In total, 490 sequences across 13 European countries (Table 1 and Table 2) were included in the genotyping analysis. Still, the data is uneven, only six countries (Italy, the UK, Spain, Sweden, France, and Germany) reported more than 10 genotyped sequences. Italy had most with 177 sequences, but 60 % were too short for reliable HEV-3 subtyping (Table 2; unclassified-3). Italy also reported the only HEV-4 strain in wastewater and earlier detections of HEV-1 in wastewater. Spain similarly had low subtyping rates, though HEV-1 and HEV-3f were observed. In other European countries, including the UK, Sweden, Germany, and France, subtype 3c dominated (Table 2). Other countries, including Serbia, Slovenia, Norway, Portugal, Russia, Switzerland, and the Netherlands, reported fewer than five sequences, making it difficult to assess HEV diversity or trends. Overall, HEV-3 was found in 87.5 % of environmental sequences, aligning with its dominance in clinical cases. Among subtyped HEV-3 strains, 3c was most common (66.0 %; 167/253), followed by 3f (27.3 %; 69/253). Nonetheless, differences between countries suggest local circulation patterns and the potential influence of regional reservoirs and practices.

**Table 2:** Genotype and subtype distribution of HEV sequences identified in environmental sources from European countries.

**Countries**	**Genotype**											**Total**^**1**^
	HEV-1	HEV-4	HEV-3 clade 1 (efg)		HEV-3 clade 2 (abchijklm)						HEV-3 unclassified	
			3e	3f	3a	3b	3c	3i	3 h	3 m		
France	–	–	–	43	–	–	58	1	1	2	1	106
Germany	–	–	–	2	–	–	18	–	–	–	3	23
Italy	49	1	–	15	–	–	4	–	–	–	108	177
Netherlands	–	–	1	1	–	–	–	–	–	–	–	2
Norway	1	–	–	–	–	–	–	–	–	–	3	4
Portugal	–	–	–	1	–	–	–	1	–	–	–	2
Russia	–	–	–	–	–	–	–	–	–	–	2	2
Serbia	–	–	–	–	–	–	–	–	–	–	2	2
Slovenia	–	–	–	–	–	–	–	–	–	–	2	2
Spain	10	–	–	4	–	–	–	–	–	–	51	65
Sweden	–	–	–	1	1	1	30	–	–	–	–	33
Switzerland	1	–	–	–	–	–	–	–	–	–	–	1
UK	–	–	6	2	1	–	57	–	–	1	4	71
**Total**	**61**	**1**	**7**	**69**	**3**	**1**	**167**	**2**	**1**	**3**	**175**	**490**

1 The analysis was based on data available until September 2025.

Detailed genotyping results for sequences from France, Germany, Italy, Spain, and the UK are provided in Supplementary Table 1. Among 5333 sequences analyzed, 1175 could not be assigned to a genotype or a HEV-3 subtype by HEV genotyping tool. The remaining sequences successfully subtyped were divided into human and animal sources, and compared with environmental sequences from each country. The results showed revealed distinct country-specific patterns (Fig. 1 and Table 3). In Spain, subtype 3f predominated across all sample types. In Germany, subtype 3c was dominant in humans, animals, and environmental samples. In Italy, subtype 3f prevailed in human and environmental samples, while both 3c and 3f were almost equally represented in animals. In the UK, subtype 3c dominated in humans and environmental samples, whereas 3e was most common in animals. France exhibited the greatest genotype and subtype diversity, with HEV-1, HEV-2, HEV-3, and HEV-4 detected in humans. Among these, subtype 3f was predominant in humans and animals, while subtype 3c was more frequently reported from environmental samples. Overall, these results highlighted regional variation and complexity in HEV circulation across Europe.

**Table 3:** Distribution of HEV genotypes and subtypes across human, animal, and environmental samples in six European countries reporting at least 20 environmental sequences.

Country	Source^2^	HEV-1	HEV-2	HEV-4	HEV-3 clade 1 (efg)			HEV-3 clade 2 (abchijklm)								Total^3^
					3e	3f	3 g	3a	3b	3c	3i	3 h	3i	3 l	3 m	
France	*Homo sapiens*	11	1	40	50	1855	–	6	1	205	1	151	–	3	33	2357
	Animals	–	–	–	3	93	–	21	–	3	–	2	–	1	–	123
	Environmental	–	–	–	–	43	–	–	–	58	1	1	–	–	2	105
UK	*Homo sapiens*	12	–	–	68	8	–	14	–	124	–	–	–	–	–	226
	Animals	–	–	–	14	–	–	–	–	1	–	–	–	–	–	15
	Environmental	–	–	–	6	2	–	1	–	57	–	–	–	–	1	67
Italy	*Homo sapiens*	1	–	2	14	52	–	1	–	30	1	1	–	–	–	102
	Animals	–	–	2	51	94	–	28	–	100	–	–	–	19	9	303
	Environmental	49	–	1	–	15	–	–	–	4	–	–	–	–	–	69
Spain	*Homo sapiens*	4	–	–	1	366	–	–	–	13	–	–	–	–	26	410
	Animals	–	–	–	1	119	–	–	–	14	–	–	–	–	11	145
	Environmental	10	–	–	–	7	–	–	–	–	–	–	–	–	–	17
Germany	*Homo sapiens*	4	–	–	14	25	–	3	–	380	–	–	–	–	3	429
	Animals	–	–	–	8	3	1	4	–	29	2	1	–	–	–	48
	Environmental	–	–	–	–	2	–	–	–	18	–	–	–	–	–	20
Sweden^1^	*Homo sapiens*	6	–	–	17	73	–	5	2	57	–	1	–	–	5	166
	Animals	–	–	–	11	113	–	5	4	2	–	–	–	–	1	136
	Environmental	–	–	–	–	1	–	1	1	30	–	–	–	–	–	33

1 Data from Sweden has been previously published [29].

2 Sequences without classified subtype information were excluded.

3 The analysis was based on data available until September 2025.

**Fig. 2:**
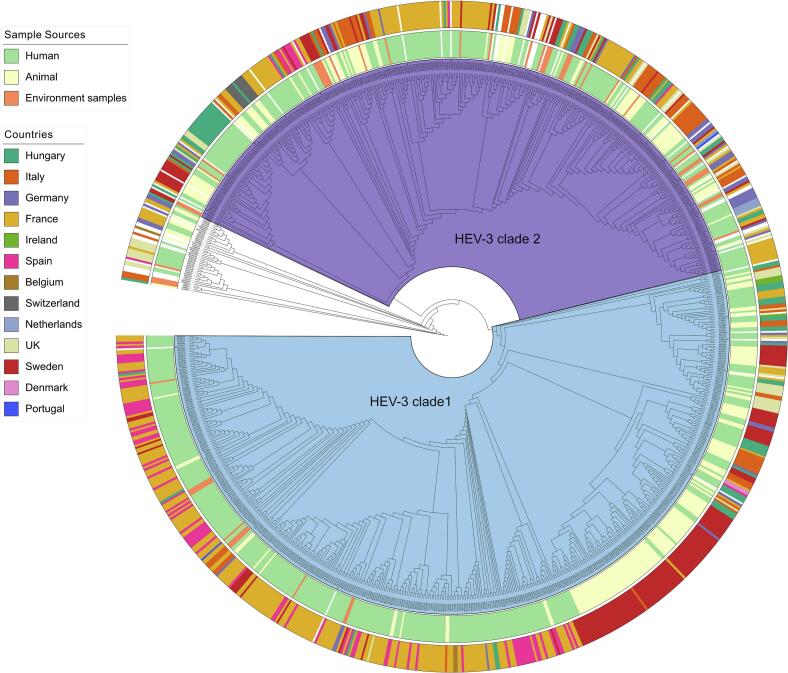
Phylogenetic tree of HEV Sequences from Europe at the partial RdRp Region. Phylogenetic tree was constructed based on the 309 nucleotides of partial HEV RdRp region using UPGMA method. A total of 957 HEV sequences were downloaded from the NCBI Database and aligned using MAFFT. The tree was generated with MEGA11. The sample source and origin country were annotated by color at iTOL software. Two major clades of HEV-3 strains are labelled, with clade 1 include HEV-3efg subtypes, while clade 2 includes HEV-3abchijklm subtypes. HEV-3ra (rabbit-associated) subtype and other HEV genotypes that outside the two major clades are not highlighted.

Furthermore, phylogenetic analysis of partial HEV RdRp sequences from European countries was performed (Fig. 2). In line with Table 2, most sequences clustered within HEV-3, forming two major clades: clade 1 (subtypes e, f, g) and clade 2 (subtypes a, b, c, h, i, j, k, l, m). Environmental sequences were distributed across both clades and showed close genetic similarity to strains from humans and animals, suggesting that environmental sources can provide important insights into transmission between human and animal reservoirs. The information on origin country was also annotated in the analysis. Some countries showed country-specific clustering, such as France in HEV-3 clade 1 and Germany in HEV-3 clade 2. Other countries displayed contrasting patterns, for example, environmental strains from Sweden were mainly located in HEV-3 clade 2, whereas strains from Swedish patients and animals clustered in HEV-3 clade 1. These findings emphasize the value of environmental sources as a complementary tool for HEV investigation, particularly in high-prevalence regions such as France.

## 4 Discussion

This review provides an updated overview of HEV presence in environmental sources across Europe, combining sequence data from open access public databases with published studies. Both infected individuals and animals carrying zoonotic HEV genotypes can shed viral particles into wastewater, surface water, soil, and other environmental matrices. In this context, environmental monitoring represents a valuable complementary tool to track the circulation of HEV among humans and animals. Our findings show that, although more than 13,000 HEV sequences are available from Europe, only a small fraction (2.4 %) originates from environmental samples, and mostly from a few countries, including Italy, the UK, Spain, France, Germany, and Sweden. This limited and uneven geographic distribution underscores substantial regional gaps in environmental HEV surveillance across Europe. Such imbalance is likely driven by differences in national surveillance strategies, availability of funding and laboratory resources, sequencing capacity, and possibly underlying variation in HEV epidemiology between regions. Phylogenetic analysis further showed that HEV strains from environmental sources are interspersed with strains from humans and various animals. Together, these findings support the integration of clinical, veterinary, and environmental data within a One Health framework to better elucidate HEV transmission and to guide effective surveillance and intervention strategies.

Our analysis showed substantial variation in HEV-3 subtypes across European countries and sample types. In some countries, such as Germany and Spain, the same dominant subtype circulated consistently among humans, animals, and environmental samples. In contrast, other countries displayed divergent patterns. For instance, in both France and Sweden, subtype 3f predominated in humans and animals, whereas subtype 3c was more frequently reported from environmental sources. This discrepancy raises new questions about HEV-3 transmission. It remains unclear whether individuals infected with different subtypes exhibit distinct fecal excretion patterns, including differences in viral load or duration of excretion. Previous studies have suggested that infections with subtype 3c are often associated with milder symptoms and lower hospitalization rates [[62], [63], [64], [65]], which could result in underrepresentation of 3c in clinical data despite its frequent detection in environmental samples, mainly wastewater. In addition, subtype-specific differences in RNA stability or persistence in environmental matrices may influence detection efficiency, potentially favoring certain subtypes in wastewater samples. In the UK, subtype 3e dominated in animals but was rarely found in humans or environmental sources. This unique circulation pattern suggests that local animal reservoirs do not fully account for the HEV strains circulating in the population. One explanation is the importation of infections through the food chain, as the UK imports large amounts of pork from European countries such as the Netherlands and Germany [66], where subtype 3c is prevalent among pig populations [67]. Given the large geographic scale and complex transmission routes in Europe, significant heterogeneity in HEV prevalence and subtype distribution exists both between and within countries. For example, a French study found consistent subtype patterns between wastewater and blood donor samples, with 3c been predominating in both samples, in the Toulouse (Southern France) and Dunkerque (Northern France) [41], while in other regions, such as Clermont-Ferrand area (Central France), subtype 3f was dominant in both clinical and wastewater samples [35].

Recent studies have explored the potential correlation between HEV detection in wastewater and clinical cases, but observed no direct correlation [26,68]. The constant detection of HEV in wastewater, despite strikingly low clinical cases reports and a high seroprevalence among blood donors [69], suggest the silent spread in the community that was missed by clinical routine diagnosis. This discrepancy is likely due to the higher proportion of asymptomatic or subclinical infections, low awareness, and underdiagnosis in clinical settings. Moreover, zoonotic sources such as swine, rodents, pets, and other animal hosts may contribute to higher genome prevalence in wastewater. These observations reveal the shortcomings of purely clinical surveillance and point to the need for broader, population-level approaches that do not depend on testing symptomatic cases. Although wastewater surveillance may have limitations as an early warning system, it has proven effective in detecting clinically relevant HEV strains missed by clinical testing [21,47]. This advantage is particularly relevant in the context of long-term epidemiological changes. For example, a shift in circulating HEV-3 subtypes, with subtype 3c gradually replacing 3f, has been reported in several European countries over the past decade [2,70,71]. Detecting such changes through clinical surveillance alone typically requires large-scale and long-term screening of patients or blood donors, which is resource intensive. Environmental surveillance therefore could offer a more efficient complement approach to identify community-level subtype shift and to inform a more targeted clinical testing.

Besides HEV-3, other genotypes have also been sporadically detected from environmental sources in Europe. HEV-1, typically associated with travel-related infections outside the EU/EEA, has been reported in three European countries, but no detection from environmental samples since 2016, despite occasional human cases still occurring. HEV-4, another zoonotic genotype more common in Asia, has been reported sporadically in Europe, mostly in France [72], but its detection in environmental waters is extremely rare, with only one report of HEV-4d in Italian wastewater and no corresponding clinical cases in the same region [21]. These findings demonstrate how environmental surveillance can capture rare genotypes that may be missed by clinical testing. Rat HEV is an emerging virus capable of causing both acute and chronic hepatitis in immunocompetent and immunocompromised individuals and has recently been linked to cases of acute hepatitis of unknown origin [73,74]. This virus has been repeatedly detected in wastewater from several European countries, often at relatively high concentrations [[22], [23], [24], [25], [26]]. Its frequent detection likely reflects rodent activity in sewage systems and continuous viral shedding. The exact transmission route for rat HEV is still unclear, but its presence in environmental samples raises the public health concerns as widespread environmental circulation may increase opportunities for indirect human exposure. These observations demonstrate substantial gaps in our understanding of rat HEV transmission, exposure pathways, and public health significance. Overall, the sporadic detection of HEV-1 and HEV-4, together with the growing prominence of rat HEV, highlights both the strengths and limitations of environmental surveillance in Europe. The scarcity of non-HEV-3 data complicates efforts to assess community transmission.

Despite the growing need for more HEV sequences from environmental sources, several technical challenges persist. Polymerase chain reaction (PCR) amplification with Sanger sequencing is the most common method for characterizing HEV. However, this approach has significant limitations in environmental surveillance. First, low viral loads and PCR inhibitors in environmental waters often cause amplification failure. Second, environmental water frequently contains mixed HEV strains. This leads to preferential amplification of dominant variants, causing biased detection. For example, rat HEV may dominate sequencing outputs and potentially obscure the detection of human HEV strains when using broad-range PCR assays in wastewater, as rat HEV is typically present at 1–2 log₁₀ higher concentrations [24]. Finally, the short amplicons (200–400 bp) offer limited phylogenetic resolution, making it difficult to differentiate closely related subtypes, such as subtype 3c and 3i.

Metagenomic sequencing and long-read sequencing are alternatives methods. Studies have successfully used these methods to identify HEV-3, HEV-4 and emerging rat HEV in wastewater [21,23,34,39,41,47]. However, several limitations hinder its routine use. Detection is concentration-dependent, and HEV RNA levels in environmental waters are often lower than other viruses like SARS-CoV-2 or norovirus, resulting in poor sequence recovery. Even in quantitative PCR (qPCR) positive samples, metagenomic sequencing may fail to detect HEV due to sample complexity and pre-amplification bias. Long-read sequencing enables longer genome recovery and better subtyping. While Nanopore sequencing has proven effective for SARS-CoV-2 wastewater surveillance [75], its use for HEV remains rare. These methods are complementary and should be selected based on available resources and research goals. Regardless of approach, improving virus concentration methods and removing environmental inhibitors is essential to enhance amplification.

Unlike SARS-CoV-2 surveillance, which has a guideline from the World Health Organization [76], environmental surveillance for HEV lacks standardized protocols across laboratories for virus concentration, RNA extraction, qPCR, and PCR amplification. This methodological variability contributes to inconsistent detection rates and differences in sequencing success between studies, limiting direct comparison of results. Moreover, HEV sequences from environmental sources remain rare and are derived from a limited number of studies. These samples are often collected at time points that do not align with available human or animal data, which restricts meaningful temporal comparisons. The wide heterogeneity of environmental matrices, including wastewater, surface water, and biosolids, further complicates cross-study comparisons. Therefore, these limitations reflect a major gap in current HEV surveillance rather than shortcomings of individual studies, and there is a need for more systematic environmental monitoring. The HEVnet initiative was established to coordinate and enhance HEV molecular typing and epidemiological investigations across human, animal, food and environmental samples [77]. While environmental samples are included within this framework, no environment-specific surveillance protocol is currently available. Developing and adopting consensus methods for environmental HEV detection would greatly improve data reliability and comparability. In addition, given the widespread collection and archiving of wastewater samples during the COVID-19 pandemic across Europe, there is a unique chance to deepen our understanding of HEV transmission by exploring these samples.

## 5 Conclusions

This study highlights both the benefits and current challenges of using environmental samples to monitor HEV transmission. Environmental surveillance, particularly through wastewater, can detect emerging variants earlier than clinical testing, guide targeted interventions, and provide unique insights into HEV circulation in Europe, complementing clinical and veterinary data under a One Health framework. Despite this potential, environmental HEV surveillance in Europe remains unevenly implemented, with large geographic gaps, limited sequencing data, and substantial methodological heterogeneity. Strengthening HEV surveillance in Europe could be achieved through (i) increasing surveillance in countries where discrepancies between environmental and clinical subtype distributions have been observed; (ii) optimizing and standardizing the surveillance workflow, including virus concentration methods, detection assays, and sequencing strategies, to improve sensitivity and comparability across studies; (iii) establishing a coordinated international initiative to support standardize protocols, expand sequencing capacity, and facilitate data sharing; (iv) retrospective analysis of archived wastewater samples collected before and during the COVID-19 pandemic represents a cost-effective opportunity to understand past HEV circulation and subtype dynamics. Altogether, these efforts would improve our knowledge of HEV transmission and bolster public health preparedness against emerging HEV threats.

Declaration of generative AI and AI assisted technologies in the writing process.

During the preparation of this work the author used ChatGPT Edu in order to check for spelling and grammar errors. After using this tool/service, the author reviewed and edited the content as needed and takes full responsibility for the content of the published article.

## CRediT authorship contribution statement

**Hao Wang:** Visualization, Validation, Supervision, Methodology, Investigation, Funding acquisition, Formal analysis, Data curation, Conceptualization, Writing – review & editing, Writing – original draft. **Marianela Patzi-Churqui:** Visualization, Software, Writing – review & editing. **Linn Dahlsten Andius:** Visualization, Validation, Writing – review & editing. **Kristina Nyström:** Resources, Writing – review & editing. **Martin Lagging:** Supervision, Funding acquisition, Writing – review & editing.

## Declaration of competing interest

The authors declare that they have no known competing financial interests or personal relationships that could have appeared to influence the work reported in this paper.

## Data Availability

Data will be made available on request.

## Appendix A Supplementary data

Supplementary material 1 Image 1

Supplementary material 2 Image 2

Supplementary material 3 Image 3
